# Effect of the Lipoxygenase Inhibitor Baicalein on Muscles in Ovariectomized Rats

**DOI:** 10.1155/2016/3703216

**Published:** 2016-12-05

**Authors:** D. Saul, J. H. Kling, R. L. Kosinsky, D. B. Hoffmann, M. Komrakova, M. Wicke, B. Menger, S. Sehmisch

**Affiliations:** ^1^Department of Trauma, Orthopedics and Reconstructive Surgery, Georg-August-University of Göttingen, Göttingen, Germany; ^2^Department of General, Visceral and Pediatric Surgery, University Medical Center Göttingen, 37075 Göttingen, Germany; ^3^Department of Animal Science, University of Göttingen, Albrecht-Thaer-Weg 3, 37075 Göttingen, Germany

## Abstract

Sarcopenia, a loss of muscle mass accompanying osteoporosis, leads to falls and fall-related injuries. Baicalein, as a phytochemical agent, has an antioxidative and anti-inflammatory effect in muscle. In this study, sixty-one female Sprague Dawley rats were divided into five groups: four groups were ovariectomized (OVX) and one control group was nonovariectomized (NON-OVX). Eight weeks after ovariectomy, three disparate concentrations (1 mg/kg body weight (BW), 10 mg/kg BW, and 100 mg/kg BW) of baicalein were applied subcutaneously daily in three OVX groups. Mm. soleus, gastrocnemius, and longissimus were extracted; their diameter, area, relation to body, and muscle weights as well as number of capillaries per fibre were recorded. In Mm. soleus and gastrocnemius, the baicalein effect (increasing number of capillaries per fibre) was proportional to the dose applied. The fibre diameters and area under baicalein treatment were significantly greater compared to OVX and NON-OVX groups. In M. longissimus, we observed a shift to type IIa fibres. Serum creatine kinase levels were significantly lower in highest baicalein concentration group. We conclude that baicalein can stimulate angiogenesis, though not fibre type-specific, in skeletal muscle and reduce the estrogen-related loss of fibre diameter and area in the skeletal muscle in rats. Therefore, a protective effect of baicalein on muscle cells can be assumed.

## 1. Introduction

Osteoporosis is one of the most common diseases in humans. Recent data assume lifelong prevalence of about 11.9% of osteoporosis in Germany [1]. Significantly increased mortality rates and decreased life quality are observed in patients who have experienced fractures. Owing to their high socioeconomic relevance and increasing incidence, apart from the estimated costs of around 5.4 billion euros a year, therapeutic alternatives need to be investigated [2].

Sarcopenia, a loss of skeletal muscle mass, emerges during the development of osteoporosis [3–6]. This results in a higher risk of falls and, consequently, fall-related injuries [7, 8]. Ninety percent of all fractures in elderly people arise from falls [9]. A variety of endocrine substances synthesised by muscle which influence the bone are reduced in sarcopenia. For instance, levels of muscle-derived humoral bone anabolic factors, which activate osteoblasts, are decreased [10]. This implies that a sarcopenic muscle is an accompaniment of osteoporosis and the occurrence of osteoporotic fractures. Additionally, sarcopenia was reported to result in increased mortality in elderly women [11]. Histologically, sarcopenia leads to a reduction in size of both muscle fibre types I and II, whereas the fast type II fibres are chiefly affected [12–14].

Former studies suggested that a low grade inflammation contributes to sarcopenia [15]; however, the detailed underlying mechanisms remain unclear. The endocrine axis between muscle and bone, consisting of insulin-like growth factor 1, interleukins (ILs) 6, 7, and 15, and osteoglycin, and vice versa for sclerostin with its muscle counterparts myostatin and osteocalcin, is impaired in osteoporosis and, therefore, in sarcopenia—they seem to be “two sides of the same coin” [16–19]. Recent studies also showed a significant loss of number, thickness, and capillarization of muscle fibres with a majority of type II fibre loss [20–22].

Therefore, therapeutic possibilities reside in pharmacological intervention which target impaired regenerative capacity, elevated reactive oxygen species production, and inflammation observed in sarcopenic muscle [23]. Several of these inflammation reactions are mediated by lipoxygenases, enzymes of arachidonic acid metabolism.

Baicalein, as a phytochemical agent, is extracted from the plant Scutellaria baicalensis Georgi. It acts as a lipoxygenase (especially cyclooxygenase (COX-I) inhibitor) and is also a potent inhibitor to 12-lipoxygenase (12-LOX) and 15-LOX and, therefore, has an antioxidative effect [24–27]. Furthermore, it inhibits nuclear factor kappa-light-chain-enhancer of activated B cells (NF-*κ*B) and suppresses the function of tumor necrosis factor-alpha 8 (TNF-*α*) and/or IL-6, all of them being mediators of inflammation cascades. An effect of baicalein on inflammatory degenerative bone diseases, such as rheumatoid arthritis, through its suppression of apoptosis and secretion of matrix metalloproteinases, has been recently described [28, 29]. Baicalein, as a common flavonoid, also inhibits eotaxin production, probably having a positive effect on asthma [30]. Additionally, 12-LOX is inhibited by baicalein, where the former increases the activity of HIF-1*α* under hypoxic conditions, causing the upregulation of the vascular endothelial growth factor, a major factor in promoting prostate cancer progression and metastasis [31].

*Proposal*. As the effect of baicalein on sarcopenia has not been clarified in previous studies, we aimed to decipher whether baicalein could reduce sarcopenic symptoms in muscles of osteoporotic rats. Outcome measures included the number and diameter of muscle fibres, the relation with type I, type IIa, and type IIb muscle fibres, and the number of capillaries in muscles (the relation of capillaries to muscle fibre). These measures were investigated in M. soleus, M. gastrocnemius, and M. longissimus. Furthermore, serum levels of calcium, magnesium, and creatine kinase, a possible biomarker for sarcopenia [32], were measured to estimate the general muscle condition.

## 2. Materials and Methods

### 2.1. Animals and Treatment

For the study, 61 female, 3-month-old Sprague Dawley rats (Winkelmann Company) were kept in cages at 20°C and at a relative humidity of 55% in Makrolon IV®. After 1 week of acclimatization, the experiments were conducted in accordance with the ethical standards of animal care and approved by the local district government (application number: G14/1530).

At 13 weeks of age, the rats underwent bilateral ovariectomy (OVX) or were left intact, as previously described [33]. Surgical procedures were carried out under ketamine/domitor anaesthesia (0.1 mL/100 g body weight (BW), intraperitoneal (i.p.)). In brief, after shaving, anaesthesia, and disinfection, the skin was incised on both sides of the lower abdomen. Adnexa were dissected, clamped, and removed before the wound was closed. After 8 weeks, osteotomy of tibia metaphysis with plate osteosynthesis was performed for different studies, according to [34]. If the standardized tibia osteotomy, performed bilaterally in all rats, would have any effect on muscles, it would be similar in all treatment groups. On the basis of previous studies [35, 36], we assumed that, at around this time point, that is, 8 weeks after OVX, the rats would have developed osteoporosis with sarcopenia. Baicalein treatments were started one day after osteotomy. For injections, baicalein (98%, Sigma-Aldrich Chemie GmbH, Munich) was dissolved in 100% dimethyl sulfoxide (DMSO), while both control groups (NON-OVX and OVX, each *n* = 10) received DMSO alone. Baicalein was injected subcutaneously at different concentrations as previously described [37, 38] (C1: 1 mg/kg BW, C2: 10 mg/kg BW, and C3: 100 mg/kg BW, resp.) in three groups with 10 animals each. The injections were administered every 24 hours for 4 weeks.

### 2.2. Tissue Isolation and Processing

The animals were sacrificed after the baicalein treatment. M. gastrocnemius, M. soleus, and M. longissimus were removed, and M. gastrocnemius and M. soleus were weighed. M. soleus was cut 4-5 cm before the foreleg. Afterwards, M. gastrocnemius and M. soleus were sectioned transversely in half across the whole muscle to make cryotome-cutting easier.

The muscles were frozen in liquid nitrogen and stored at −80°C. Subsequently, 12 *µ*m thick frozen sections were prepared by cutting the muscle samples orthogonally with a cryotome (CM 1900; Leica Microsystems).

### 2.3. Staining

**Figure 1:**
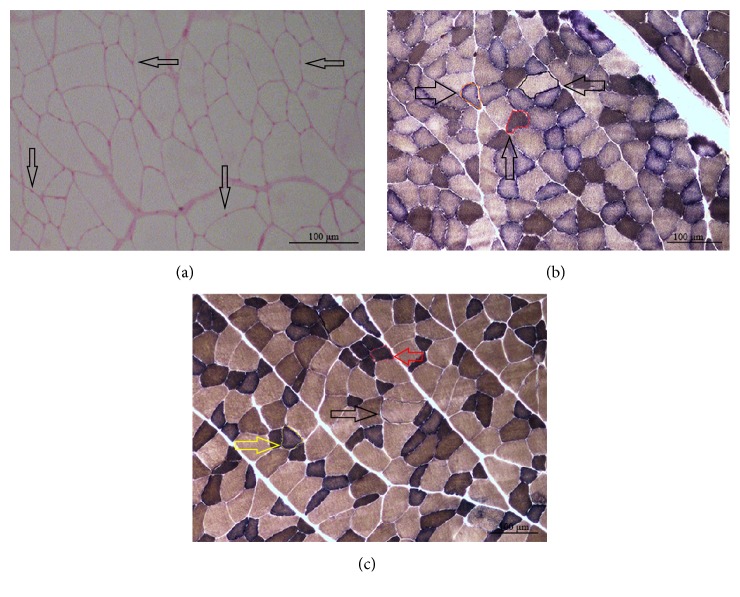
PAS and ATPase staining of M. longissimus ((a) and (c)) and M. gastrocnemius (b). The PAS staining of M. longissimus shows capillaries marked by arrows (a). In ATPase staining, type I fibres are enclosed in red, IIa fibres in black, and IIb fibres in orange in M. gastrocnemius, whereas example fibres are marked by black arrows (b); in M. longissimus, type I fibres are marked by a red arrow (surrounded by a red shape), type IIa fibres with a black arrow (surrounded by a black shape), and IIb fibres with a yellow arrow (surrounded by a yellow shape) (c) (100x magnification).

To identify the distribution and changes in types I and II fibres, ATPase staining was carried out on muscle sections, as described by Horák [39]. Moreover, Periodic acid-Schiff (PAS) staining to visualize capillaries in muscle and subsequent counting was performed according to the method described by Andersen et al. [40, 41]. Representative stainings are shown in Figure 1.

For capillary counting, two sections of each muscle in PAS staining were chosen and, in a 0.25 mm^2^ square, every capillary and muscle fibre were counted, which taken together delivered the ratio of capillary/muscle fibre.

For fibre measurements, in three different image sections of each muscle in ATPase staining, 30 type I, 30 type IIa, and 30 type IIb fibres were edged and area and diameter were determined by the program NIS-Elements AR 4.0 (Nikon Instruments Europe, Amsterdam, Netherlands). After that, in 1 mm^2^ fibre, types were counted and their relation calculated.

### 2.4. Serum Values of Creatine Kinase, Calcium, and Magnesium

After the rats were sacrificed, blood samples of 0.5 mL were collected and analysed with the creatine kinase assay using a c16000 analyser (Abbott, Wiesbaden, Germany) for quantitative measurement of serum values. Calcium was measured with the same analyser at 660 nm using Arsenazo III dye and magnesium at 572 nm after the calcium was complexed and eliminated with a chelating agent so as to not interfere with the measurements.

### 2.5. Statistics

Statistical analyses were conducted using GraphPad Prism (version 5.04, GraphPad Software, Inc., San Diego, CA). For detecting differences between the different groups, one-way ANOVA (*F* test, *α* = 0.05) was applied. Differences between individual means were estimated using Tukey's test (*α* = 0.05). In all figures, the mean values and the standard error of the mean (SEM) are displayed.

## 3. Results

### 3.1. Rat Characteristics

**Figure 2:**
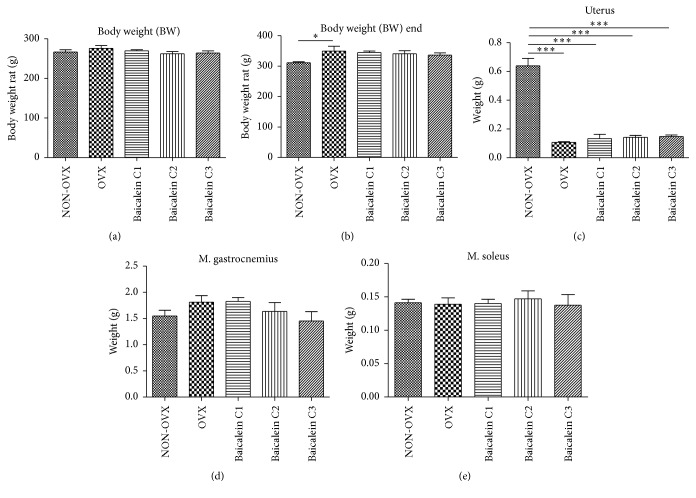
Body weight in the beginning (a) and end (b) of trial, weight of uteri (c), M. gastrocnemius (d), and M. soleus (e). The body weight of rats was monitored on the days of delivery ((a), *n* = 48) and sacrifice ((b), *n* = 48). Cohorts included nonovariectomized (NON-OVX), ovariectomized (OXV) rats, and animals treated with different baicalein concentrations (C1: 1 mg/kg BW, C2: 10 mg/kg BW, and C3: 100 mg/kg BW). The uterus weight in NON-OVX rats was significantly greater than that in the other groups ((c), *n* = 48). M. gastrocnemius and M. soleus were weighed after decapitation and no significant differences could be measured, while in the former a higher concentration of baicalein showed a tendency towards lower muscle weight ((d), *n* = 46; (e), *n* = 42). ^*∗*^ *p* < 0.05 and ^*∗∗∗*^ *p* < 0.001 indicate significant difference.

To test the effects of ovariectomy and different concentrations of baicalein on body weight, the rats were weighed on the day of receipt (Figure 2(a)) and at postmortem (Figure 2(b)).

There was no significant difference between groups in body weight on the day of receipt. However, a significant difference in postmortem weight was noted between the NON-OVX and OVX groups. The NON-OVX group was significantly lighter than the OVX group.

The postmortem weights of M. gastrocnemius (Figure 2(d)) and M. soleus (Figure 2(e)) were not statistically different; the weight of M. longissimus was not detected.

To test the effects of ovariectomy on the uterus, the uteri were extracted and weighed (Figure 2(c)). The uteri of NON-OVX rats weighed approximately 0.6 g and were significantly heavier than those collected from the other groups (on average, 0.1–0.2 g). These findings indicate successful ovariectomy with resulting atrophy of the uterus in all OVX groups.

### 3.2. Ratio of Capillary to Muscle Fibre

**Figure 3:**
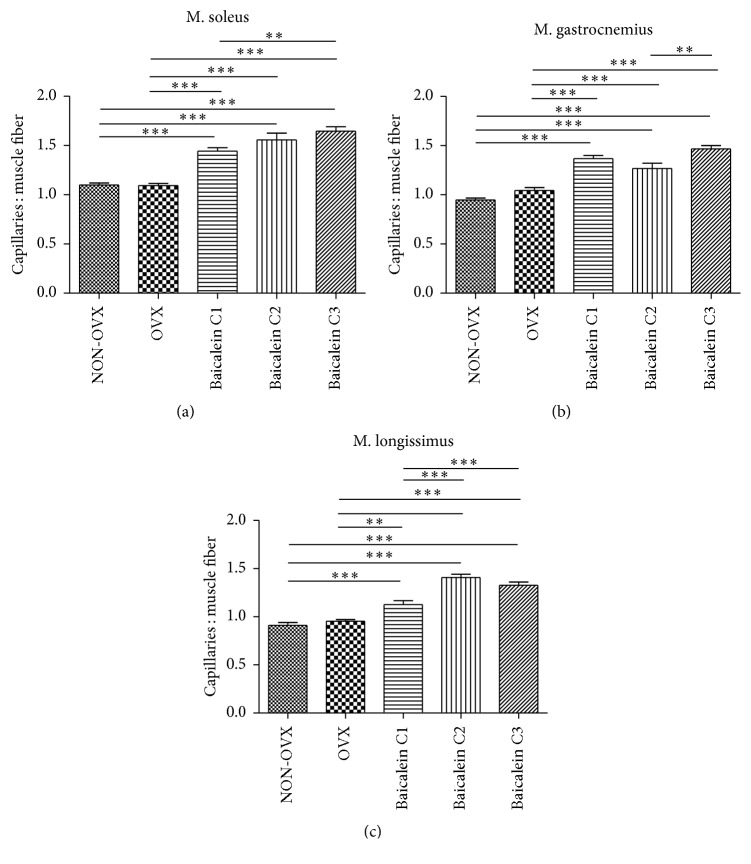
Number of capillaries per single muscle fibre in M. soleus, M. gastrocnemius, and M. longissimus. The numbers of capillaries per muscle fibre in M. soleus ((a), *n* = 92), M. gastrocnemius ((b), *n* = 92), and M. longissimus ((c), *n* = 91) per 0.5 mm^2^ were counted; groups treated with baicalein showed a significantly higher number of capillaries per muscle fibre when compared with the untreated groups. The group treated with the highest concentration of baicalein had the most number of capillaries per fibre. ^*∗∗*^ *p* < 0.01 and ^*∗∗∗*^ *p* < 0.001 indicate significant difference.

In a previous study, aging was correlated with a decrease in the number of capillaries per muscle fibre [21]. Since sarcopenia is associated with lower skeletal muscle capillarization [42], the effect of baicalein on muscle capillarization was interesting; however, the effect of baicalein on muscle capillarization has not been described yet. We analysed the number of capillaries per 0.5 mm^2^ muscle tissue in M. soleus (3A), M. gastrocnemius (Figure 3(b)), and M. longissimus (Figure 3(c)).

Although we could not see differences between NON-OVX and OVX animals, rats treated with baicalein showed significantly higher numbers of capillaries per muscle fibre when compared with the untreated groups. On average, the rate of capillarization could be increased between 20% and 40% when rats were treated with baicalein. Moreover, the group treated with the highest concentration of baicalein (C3) showed significantly more capillaries per fibre when compared with the group treated with the lowest concentration (C1).

### 3.3. Diameter and Fibre Area of M. Soleus and Their Relation to Muscle and Body Weight

After ATPase staining, muscle fibres were analysed for their diameter and area. In total, 90 fibres of each type for each sample were assessed, and 90% of all cells were type I; therefore, no further differentiation was performed.

To assess characteristics of sarcopenia, we determined the diameters and areas of the muscle fibres.

**Figure 4:**
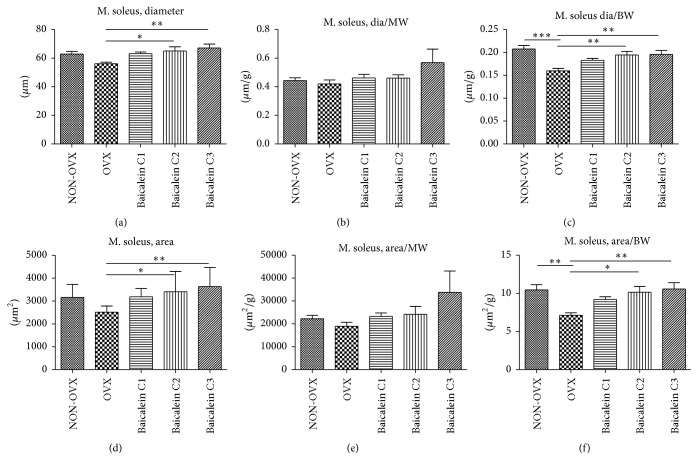
M. soleus fibre diameter and area and their relation to body weight. The diameter of single fibres was increased in each baicalein C2 and baicalein C3 group versus the OVX group ((a), *n* = 46), while OVX does not differ significantly from C1 or NON-OVX. Muscle fibre areas were measured after preparing muscle sections. Although the area in the OVX groups was decreased when compared with that in NON-OVX animals, baicalein treatment could prevent this effect ((d), *n* = 46). As for the relation of diameter to muscle weight, no significant differences could be seen ((b), *n* = 41). Compared to body weight, the fibre diameters in NON-OVX rats were significantly greater when compared with those in OVX rats. Besides, fibre to body weight ratio was higher in the baicalein C2 and C3 animals versus OVX animals ((c), *n* = 46). NON-OVX versus OVX animals had no differences in area/muscle weight ratio ((e), *n* = 40) but a significantly greater area to body weight ratio; the same was noted for each baicalein C2 and baicalein C3 group versus the OVX group ((f), *n* = 46). ^*∗*^ *p* < 0.05, ^*∗∗*^ *p* < 0.01, and ^*∗∗∗*^ *p* < 0.001 indicate significant difference.

For M. soleus, fibre diameter was determined and analysed in relation to body weight. Although the diameter in NON-OVX rats was on average 63 *µ*m, it was reduced in the OVX cohort (approximately 57 *µ*m; Figure 4(a)). Compared with OVX group, baicalein C2- and C3-treated groups possessed single fibres with significantly increased diameters (65–68 *µ*m). Muscle fibre areas were calculated after preparing muscle sections (Figure 4(b)); 90 fibres per muscle were assessed. The OVX group showed a decrease in area (2,511 *µ*m^2^) when compared with the NON-OVX control group (3,161 *µ*m^2^). Upon comparing the baicalein-treated groups to OVX groups, significantly greater areas were found in the C2 and C3 groups (3,408 *µ*m^2^ and 3,630 *µ*m^2^, resp.). Effects of baicalein on area and diameter were confirmed when observing the results in relation to body weight (Figures 4(c) and 4(d)). Assessment of single muscle weight in relation to muscle diameter showed no significant differences (data not shown).

When relating muscle fibre diameter and area to muscle weight, no significant differences, but similar tendencies to the relation to body weight, were seen. This is perhaps because of relatively low muscle weight, which could lead to measurement inaccuracy (Figures 4(b) and 4(e)).

### 3.4. Diameter and Fibre Area of M. Gastrocnemius and Their Relation to Muscle and Body Weight

**Figure 5:**
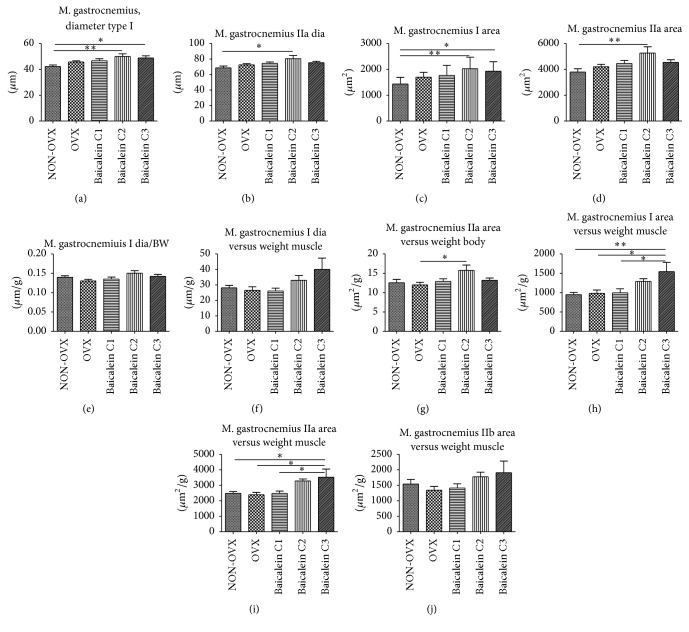
Diameter and area of M. gastrocnemius fibre types I and IIa and their relation to muscle and body weights. The diameter of single type I fibres was significantly greater in the baicalein C2 and baicalein C3 groups compared with the NON-OVX group ((a), *n* = 47). Type IIa fibres showed a greater diameter in the baicalein C2 group compared with the NON-OVX group ((b), *n* = 47). The area of type I muscle fibres was significantly greater in the baicalein C2 group and the baicalein C3 group versus the NON-OVX group ((c), *n* = 47), whereas the area of type IIa muscle fibres was significantly greater in the baicalein C2 group versus the NON-OVX group ((d), *n* = 47). No significant differences were noted in the fibre to body weight ratio in type I fibres of M. gastrocnemius ((e), *n* = 47). No significant differences in type I fibre diameter in relation to muscle weight could be detected ((f), *n* = 46). The only significant difference regarding the area to body weight ratio was between the baicalein C2 group and the OVX group, where the former had the greater area to body weight ratio ((g), *n* = 47). Baicalein C3-treated rats had a significantly higher muscle fibre area to muscle weight than the NON-OVX, OVX, and baicalein C1-treated rats ((h), *n* = 46). For type IIa fibres, significant differences in area to single muscle weight index were noted between the NON-OVX group and baicalein C3 group and between OVX group and the baicalein C3 group as in baicalein C1 group and baicalein C3 group ((i), *n* = 46). For type IIb fibres, no differences in area to single muscle weight index were recorded ((j), *n* = 46). ^*∗*^ *p* < 0.05 and ^*∗∗*^ *p* < 0.01 indicate significant difference.

In M. gastrocnemius, the distribution of different fibre types is very heterogeneous. We assessed the three fibre types—I, IIa, and IIb—for their diameter and area. Type I fibres showed an increased diameter of approximately 20% when treated with baicalein compared with the NON-OVX group (Figure 5(a)). A similar effect could be observed in type IIa fibres, with the strongest effect in the C2 group (Figure 5(b)). By contrast, type IIb fibres showed no significant differences among all groups (data not shown).

Regarding the size of the area of different fibre types, in type I (Figure 5(c)), compared with the NON-OVX control group, all OVX cohorts (treated and untreated) showed an increase in fibre area. Compared with the NON-OVX group, OVX rats showed an increase in area of 18%, and baicalein-treated animals showed an increase of up to 41%. C2 cohort showed the highest effect, but only C2 and C3 were statistically significant. A similar tendency was observed in type IIa fibres; however, only C2 group possessed a significantly greater area (Figure 5(d)). No significant differences were observed in the area of single type IIb muscle fibres (data not shown).

Thereafter, we calculated the diameter to body weight ratio and found no significant differences in types I (Figure 5(e)), IIa, and IIb fibres (data not shown). Similar results were obtained when relating diameter to muscle weight for types I (Figure 5(f)), IIa, and IIb fibres (data not shown), where no significant differences could be determined.

Moreover, we calculated the relation of fibre area to body weight. In type IIa fibres, we found a slight increase in M. gastrocnemius area in group C2 when compared with OVX rats; however, other groups did not show significant differences (Figure 5(g)). Overall findings for types I and IIb fibres indicated no striking differences between the groups (data not shown).

When relating area to muscle weight, we found significant differences in type I fibres, where NON-OVX, OVX, and C1 groups did not show significant differences (Figure 5(h)). However, when comparing their values to those of the C3 group, baicalein C3 treatment could increase the area by approximately 60%. Similar effects were observed for type IIa fibres, where the highest baicalein concentration increased the area by approximately 44% (Figure 5(i)). In IIb fibres, a similar tendency was observed; however, the differences were not significant (Figure 5(j)).

### 3.5. Diameter and Fibre Area of M. Longissimus and Their Relation to Body Weight

**Figure 6:**
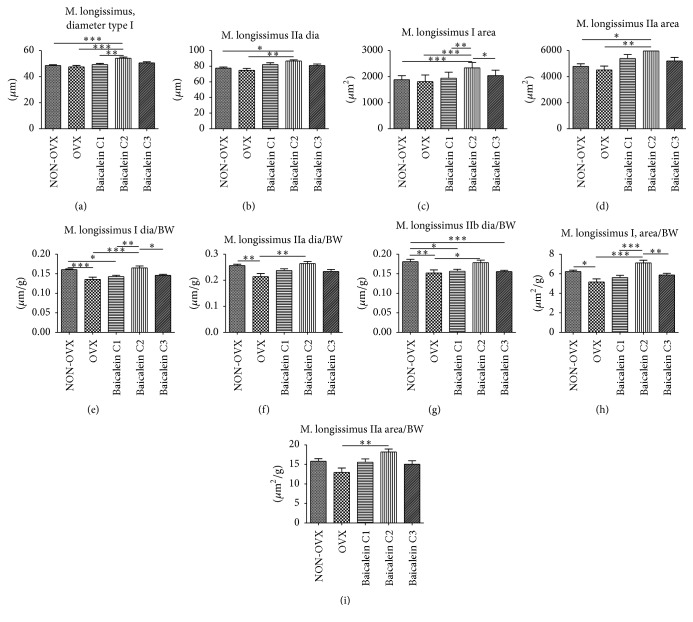
Diameter and area of M. longissimus fibre types I and IIa and their relation to muscle weight and body weights. A significantly greater fibre type I size was noted in the C2 group than in the NON-OVX, the OVX group, and the C1 group ((a), *n* = 46). A significantly greater fibre type IIa size was recorded for the C2 group compared with the NON-OVX and OVX groups ((b), *n* = 46). Significant differences in the type I fibre area could be detected when the C2 group was compared with the NON-OVX and OVX groups, C1 group, and C3 group ((c), *n* = 46). The fibre IIa area was significantly greater in the C2 group when compared with the NON-OVX and OVX groups ((d), *n* = 46). Statistically relevant differences in diameter to body weight ratio were noted between the following groups. For type I fibres: NON-OVX versus OVX and the C1 group; OVX versus C2 group, C1 versus C2 group; and C2 versus C3 group ((e), *n* = 46). For type IIa fibres, a significant difference was noted between the NON-OVX and OVX and between the OVX and C2 groups ((f), *n* = 46). The OVX group showed a significantly less fibre to body weight index when compared with the NON-OVX group and C2 group. Moreover, the NON-OVX group showed a significantly higher index than the C1 and the C3 groups ((g), *n* = 46). A significantly lower fibre area to body weight index was noted in the OVX group when compared with the NON-OVX and C2 groups. C2 group showed a higher fibre area to body weight index when compared with the OVX, C1, and C3 groups ((h), *n* = 46). C2 group, when compared with the OVX group, showed a significantly higher fibre area to body weight index for the IIa muscle fibres ((i), *n* = 46). ^*∗*^ *p* < 0.05, ^*∗∗*^ *p* < 0.01, and ^*∗∗∗*^ *p* < 0.001 indicate significant difference.

Comparable with M. gastrocnemius, the three fibre types could also be differentiated for M. longissimus. When observing the diameter in M. longissimus type I fibres, we did not detect significant differences between NON-OVX and OVX rats; however, high doses of baicalein (C2) could raise the diameter by approximately 13% (Figure 6(a)). When comparing the effects of different baicalein concentrations, the strongest effects were recorded in the C2 group.

Similar effects were observed in type IIa fibres, where baicalein C2 could increase the diameter to 86.7 *µ*m when compared with 77.6 *µ*m in NON-OVX and 74.8 *µ*m in OVX cohorts (Figure 6(b)). The IIb fibre analysis showed no significant differences among the different groups (data not shown).

We measured the area of muscle fibres types I and IIa and, analogous to the diameter, similar values were obtained for NON-OVX and OVX animals (Figures 6(c) and 6(d)). By contrast, baicalein treatment could significantly increase the muscle fibre area. While all baicalein concentrations increased the area compared with the control animals, C2 rats showed the highest values. Type IIb fibres showed no significant differences among the cohorts (data not shown).

Unlike the analysis of M. soleus and gastrocnemius, we related the fibre diameter and area in M. longissimus to animal body weight because single muscle weight was not detected. Type I fibre diameter was significantly decreased after ovariectomy (Figure 6(e)); however, after baicalein treatment (C2), this effect could be rescued. The same phenomenon was observed for types IIa (Figure 6(f)) and IIb (Figure 6(g)) fibres.

When relating the areas of types I and IIa fibres to body weight, a similar decrease could be observed in OVX rats when compared with the NON-OVX control group (Figures 6(h) and 6(i)). When treating the animals with low (C1) or high (C3) baicalein concentrations, the effect of the ovariectomy was dampened; however, fibre areas were lower than those in the NON-OVX cohort. In contrast, medium baicalein concentrations (C2) could not only rescue the consequences of the ovariectomy but also increase the fibre areas when compared with the NON-OVX rats. For IIb fibres, no significant differences between the groups could be measured (data not shown).

### 3.6. Relations of Different M. Longissimus Fibre Types

**Figure 7:**
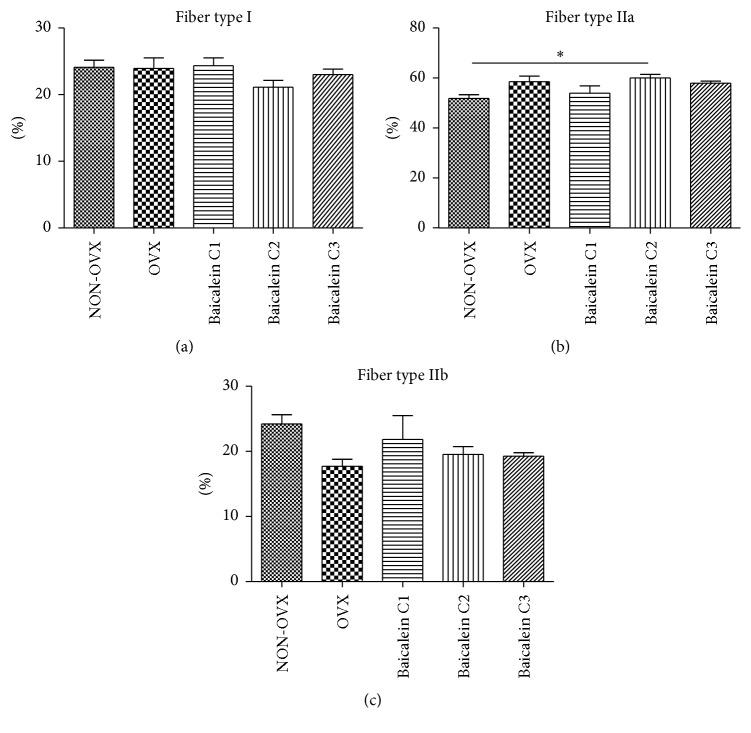
Rate of M. longissimus fibre types I, IIa, and IIb. In M. longissimus, the distribution of fibre type I was not significantly different among the groups ((a), *n* = 46). Fibre type IIa was significantly more or expanded in the C2 group versus the NON-OVX group ((b), *n* = 46). A significant increase in IIb muscle fibres was seen in the NON-OVX group when compared with the C2 group ((c), *n* = 46). ^*∗*^ *p* < 0.05 indicates significant difference.

Finally, the proportions of different fibre types (type I, IIa, and IIb) were assessed in M. longissimus (Figure 7). In general, no striking differences were observed among the different groups. The strongest effects were noted in animals treated with the medium concentration of baicalein (C2). Notably, when comparing the NON-OVX and C2 cohorts, we detected that C2 rats tended to have less type I and type IIb fibres but contained significantly more type IIa fibres.

In M. soleus and M. gastrocnemius, fibre type distribution was not determined.

### 3.7. Analysis of Serum Parameters

**Figure 8:**
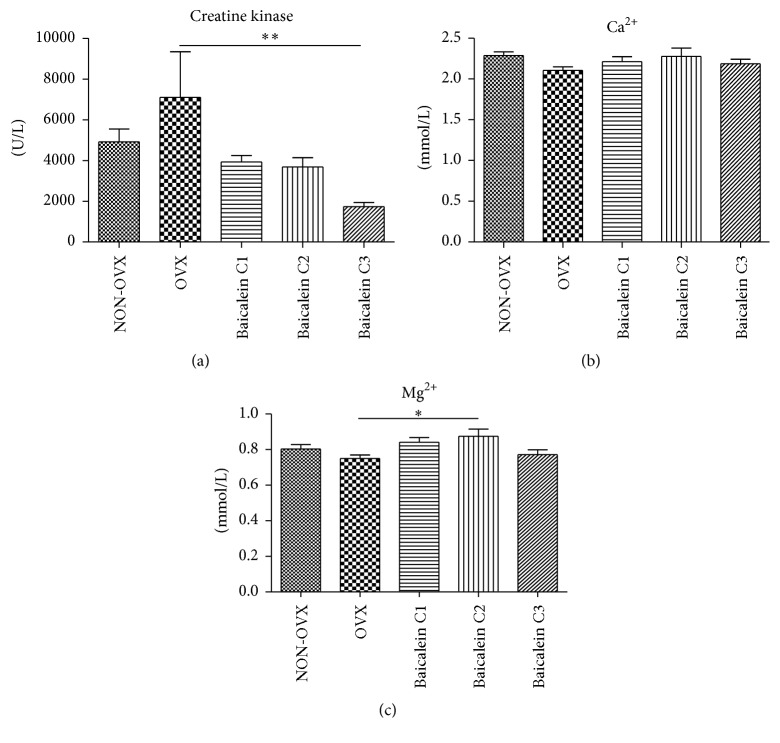
Serum creatine kinase, calcium, and magnesium levels. Compared with the OVX group, the C3 group had a significantly lower creatine kinase level ((a), *n* = 48), whereas the calcium values were not statistically significant ((b), *n* = 48). The C2 group showed a significantly higher level of magnesium when compared with the OVX group ((c), *n* = 48). ^*∗*^ *p* < 0.05 and ^*∗∗*^ *p* < 0.01 indicate significant difference.

Creatine kinase (U/L), calcium (Ca^2+^; mmol/L), and magnesium (Mg^2+^; mmol/L) levels were measured in the groups. Creatine kinase levels were significantly lower in the C3 group than in the OVX group (Figure 8(a)). The calcium levels were not considerably different across groups (Figure 8(b)). A higher serum magnesium value could be detected in the C2 group when compared with the OVX group (Figure 8(c)).

## 4. Discussion

Sprague Dawley rats are a valuable model for the induction of osteoporosis after hormonal depletion via ovariectomy [43, 44]. Here, the rats were 13 weeks old at the time of ovariectomy, which is reportedly acceptable for the osteoporosis model [45, 46]. Furthermore, Francisco et al. compared the effects of ovariectomy in rats of different ages and found no related differences in bone mineral response [47]. The effects on muscle in rats seem to be similar to those in humans: they develop sarcopenia [48, 49]. To determine the effects of baicalein on sarcopenia, baicalein was applied in OVX rats.

Independent of baicalein treatment, body weight of the rats after ovariectomy was significantly greater for the OVX (control) group than for the NON-OVX group. Application of baicalein could partially rescue the effect of estrogen deficiency in muscle. Interestingly, in M. gastrocnemius, an increasing dose of baicalein led to slightly reduced muscle weight, supporting the hypothesis of estrogen deficiency-induced muscle degeneration. However, the reduced muscle weight of M. gastrocnemius could also be attributed to the baicalein-induced inhibition of the nonphysiological degeneration of muscle, a finding reported in literature describing the anti-inflammatory and antifibrotic effects of baicalein [50].

As reported previously, estrogen has an angioproliferative effect on skeletal muscle [51, 52] and a loss of capillaries by 25% could be observed in sarcopenia because of aging [21, 53]. Notably, we could show that baicalein leads to a significant gain in the capillary to muscle fibre ratio in all baicalein-treated groups. In M. soleus, baicalein treatment showed a dose-dependent increase in the number of capillaries per muscle fibre. Similar results could also be seen in M. gastrocnemius and in M. longissimus, where the 10 mg/kg BW concentration evinced the greatest effect on capillarization.

The expected loss of capillaries under estrogen deficiency could not be observed when treated with baicalein. The hypothesis that baicalein, applied subcutaneously, stimulates angiogenesis in skeletal muscle despite estrogen deficiency was proven. Nonetheless, there were no significant differences in capillary to muscle fibre ratio after OVX, which might be due to insufficient trial duration, which was too short to reveal the decapillarization in sarcopenic muscle, as seen in other rat models [53].

We purported that decreased muscle diameter due to estrogen loss, as described in rats [53–56] and humans [57], could be restored by baicalein. Indeed, the baicalein C2 and C3 groups had significantly increased diameters of M. soleus, a finding similar to that for body weight. These findings support our hypothesis that baicalein counteracts muscle atrophy at high concentrations.

In all three fibre types (I, IIa, and IIb) in M. gastrocnemius we saw a tendency of all baicalein-treated groups to have increased fibre diameters. Interestingly, the group treated with baicalein C2 concentration showed the greatest fibre diameters.

When analysed in detail, the diameter to body weight ratio showed that types I, IIa, and IIb fibres in almost all baicalein groups were slightly increased. We also found a tendency for greater diameter per body weight values in the NON-OVX group when compared with the OVX group. Overall, baicalein appears to induce hypertrophy in the estrogen-deficient skeletal muscle.

In M. longissimus, the development of sarcopenia after ovariectomy, resulting in estrogen deficiency, which leads to reduced muscle fibre diameter is supported. The baicalein C2 group showed the highest fibre diameters.

Altogether, the expected atrophy of skeletal muscle fibres, indicated by the decline in fibre diameter [21], appears to be rescued by baicalein. The gain of IIa fibre diameter is noteworthy, considering that the diameters are typically affected in sarcopenia [53]. The fact that lipoxygenase inhibitors could counteract muscle atrophy has already been shown in clinical trials [58].

In M. longissimus, the increase in types I and IIa fibre areas suggests that baicalein can counteract the estrogen-dependent atrophy. In M. gastrocnemius, the increase of type I fibre area suggests the same, especially at the C2 concentration. Nonetheless, this gain in area and diameter can also be attributed to the storage of fat and water [59]. Moreover, estrogen deficiency can lead to an increase in noncontractile muscle fibres that can hardly be differentiated from normal muscle tissue [60]. Whether this is part of a baicalein-associated mechanism remains elusive. Microscopically, no greater fat deposits or lipid content could be observed. In affecting muscle fibre types I, IIa, and IIb, baicalein appears to react with oxidative- and glycolytic-working muscle groups.

In M. longissimus, significantly increased proportions of fast IIa but not type I fibres were observed in the baicalein C2 group. This finding disagrees with the decline in fast fibres, the described “fast-to-slow fibre type shift,” which is characteristic for muscle atrophy and wasting [61].

In conclusion, these results suggest a positive, though not fibre type-specific, effect of baicalein on estrogen-deficient muscles in rats. Our hypothesis that baicalein promotes angiogenesis in muscle could be confirmed. Whereas in M. gastrocnemius and M. soleus the number of capillaries in muscle was proportional to the dose of baicalein, in M. longissimus, the medium baicalein concentration (10 mg/kg BW) showed the greatest effect. The results of muscle diameter and area also showed a positive baicalein effect in comparison with the control groups. Moreover, regarding fibre ratios, a gain in IIa fibres was seen in M. longissimus, which contradicts the “fast-to-slow fibre type shift.”

As observed in human studies, there was no difference between sarcopenic and control groups regarding calcium and magnesium levels [62, 63].

Serum concentration of creatine kinase, a clinical marker of muscle state [32, 64], in baicalein C3 group was found to be significantly decreased. This highlights a positive effect of baicalein on skeletal muscle.

Investigating molecular mechanisms of baicalein treatment was not within the scope of this descriptive study, but Bhattacharya et al. showed that skeletal muscle atrophy (during aging and neurodegenerative diseases) can be diminished with inhibition of 12-LOX and 15-LOX, while the activation of this pathway led to increased NADPH oxidase activity, protein ubiquitination, and proteolytic degradation. They also demonstrated that baicalein reduces denervation-induced muscle atrophy in wild type but not in 12-LOX and 15-LOX knockout mice. Therefore, baicalein chiefly operates through this pathway and is not arbitrated through the reduction of inflammatory response. Possibly the main effect of baicalein in this study could be observed because of the above-mentioned 12-LOX and 15-LOX pathway inhibiting NADPH oxidase activity and protein ubiquitination as well as “ubiquitin-proteasome-mediated proteolytic degradation” [65]. Another study by Wang et al. [66] demonstrates the multiple signaling pathways in which baicalein is involved (e.g., AKT/mTOR, ERK 1/2, NF-*κ*B, and calcineurin).

The aim of this study was to elucidate whether baicalein, in different doses, applied subcutaneously, could neutralize the estrogen-induced loss of muscle and atrophy in the skeletal muscle of rats. We found that baicalein has a positive effect on estrogen-dependent atrophy of skeletal muscle.

We injected baicalein subcutaneously, which resulted in necrotic lesions at the injection sites. This could be seen in baicalein and not in DMSO-treated rats. This inflammatory effect has not been reported so far but did not affect overall health, as was seen by the analyses of body weight, food intake, and general conditions (clean coat, absence of porphyrin around eyes, etc.). The inflammatory processes were not part of this study and we could not confirm an anti-inflammatory effect after s.c. injection of baicalein as it was shown after experimental traumatic brain injury [67]. Therefore, it is advisable to use an alternative route of administration, such as orally [50, 68] or intravenously [69].

## 5. Limitations

To emphasize differences between baicalein and OVX groups, trial duration could be elongated and more groups could provide better conditions to find the ideal dose and route of baicalein administration. Moreover, a healthy NON-OVX group should be included. As we only investigated skeletal muscle, we cannot exclude systemic effects. No fever or severe infections occurred throughout the experiments. However, the development of the necrotic lesions at the injection sites should be taken into consideration. Smooth muscles and cardiomyocytes as well as infection parameters, such as CRP, IL-6, and procalcitonin, could be further examined to rule out effects on these tissues [70].

To exclude the fact that gains in area and diameter are attributed to fat and water retention, biochemical analyses of muscle fibres would be useful. Additionally, there could be a possible effect of osteotomy on muscle tissue, so these experiments could be repeated without this operative procedure; however, this effect would exist in all rats.

Different models of muscle inflammation could also be used to further investigate the anti-inflammatory effect of baicalein. In addition, the bone of rats and its biomechanical and histomorphometric composition could also be evaluated, as recently done by Li et al. [71], but were not part of this study.

Detection of the molecular mechanism underlying the effects of baicalein in rats was not part of this descriptive study. Therefore, further examinations including* in vitro* approaches should be performed.

Baicalein increased capillaries per muscle fibre but not total muscle weight. This discrepancy could be explained by baicalein-induced inhibition of the nonphysiological degeneration of muscle due to the anti-inflammatory, antifibrotic [50, 72], and antioxidant [66, 73] effects of baicalein but should be further examined.

## Abbreviations

AKT/PKB: Protein kinase b
ATP: Adenosine triphosphate
BW: Body weight
COX: Cyclooxygenase
CRP: C-reactive protein
DMSO: Dimethyl sulfoxide
ERK 1/2: Extracellular signal-regulated kinase 1/2
HIF-1*α*: Hypoxia-inducible factor 1-alpha
IGF-1: Insulin-like growth factor 1
IL: Interleukin
IL-6: Interleukin-6
I.P.: Intraperitoneal
LOX: Lipoxygenase
mTOR: Mechanistic target of rapamycin
NADPH: Nicotinamide adenine dinucleotide phosphate
NF-*κ*B: Nuclear factor kappa-light-chain-enhancer of activated B cells
NON-OVX: Nonovariectomy
OVX: Ovariectomy
PAS: Periodic acid-Schiff
SEM: Standard error of the mean
TNF-*α*: Tumor necrosis factor-alpha 8
VEGF: Vascular endothelial growth factor.
